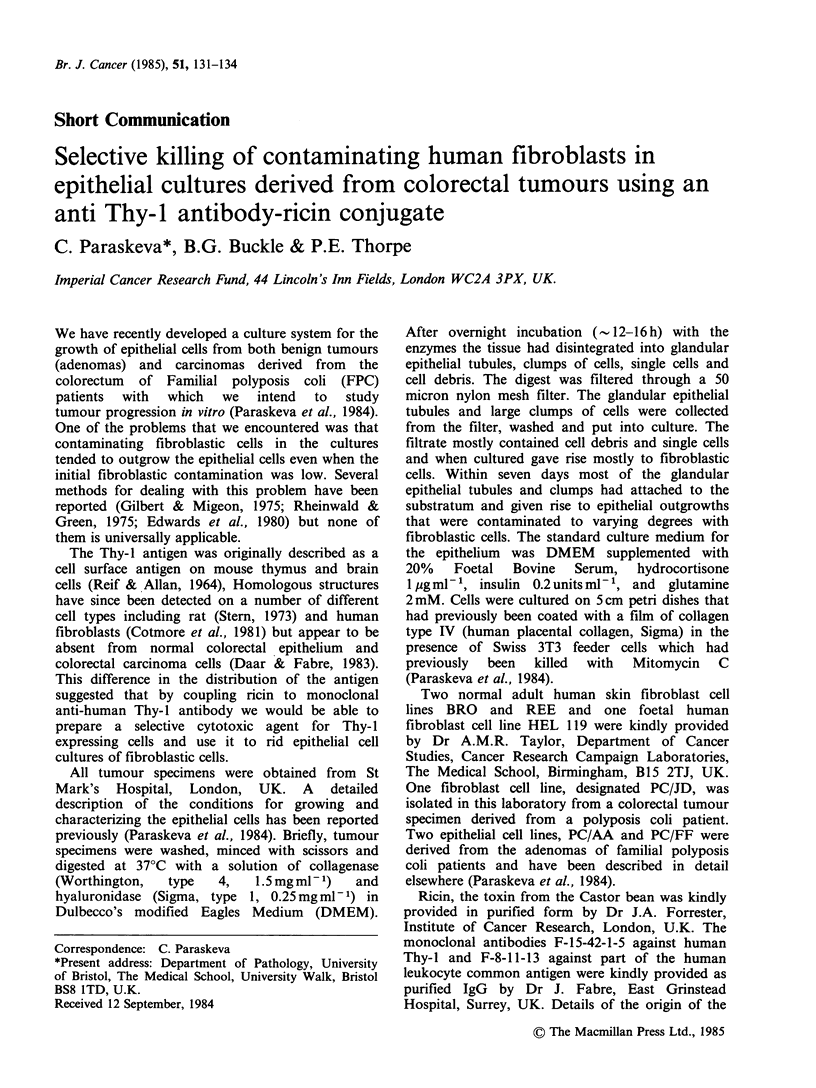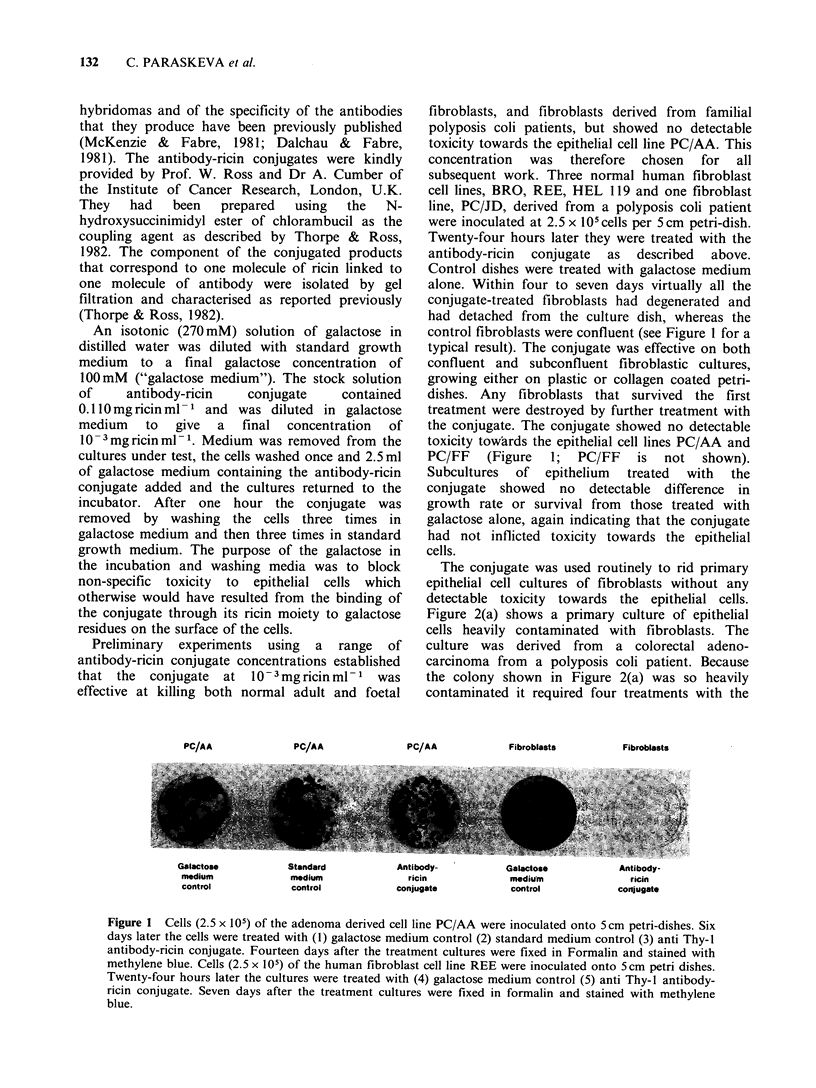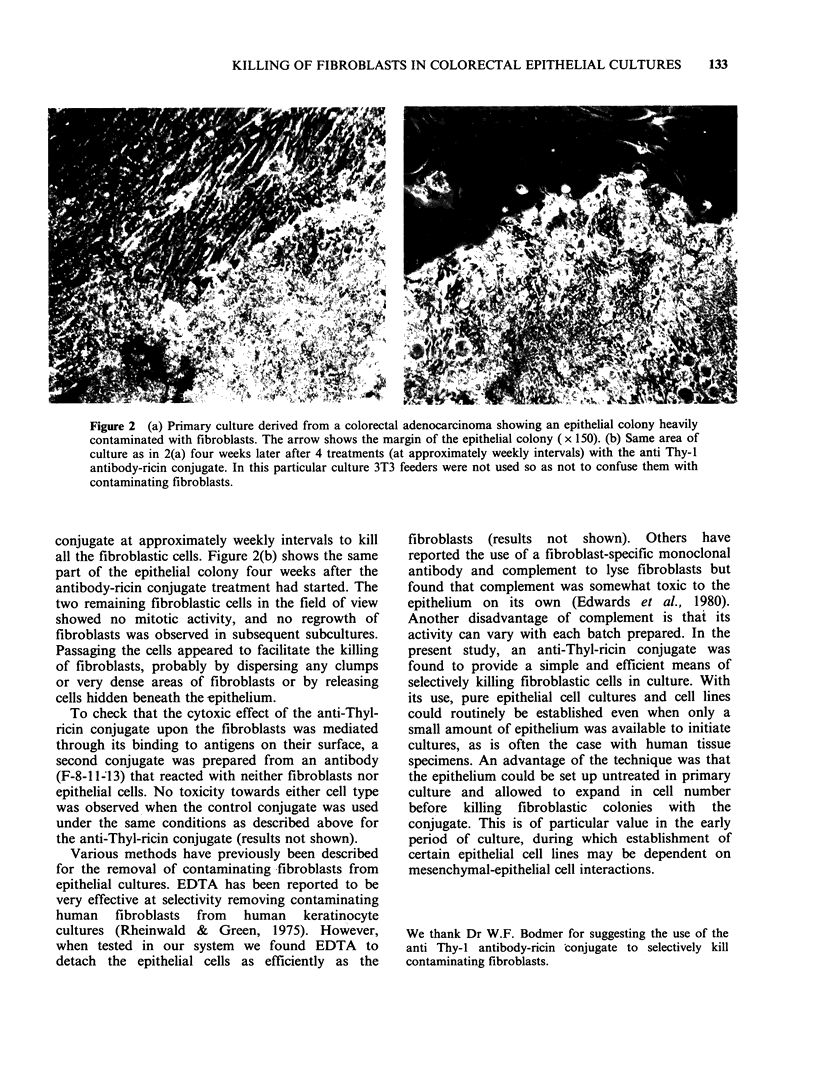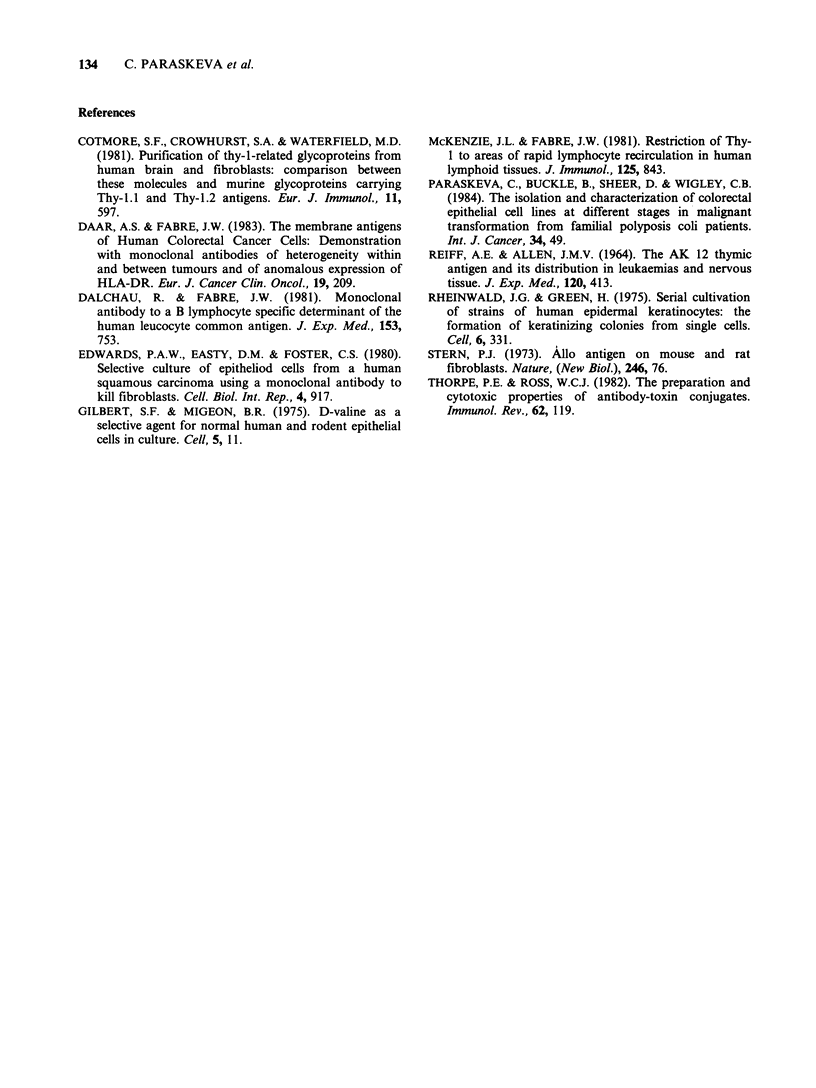# Selective killing of contaminating human fibroblasts in epithelial cultures derived from colorectal tumours using an anti Thy-1 antibody-ricin conjugate.

**DOI:** 10.1038/bjc.1985.19

**Published:** 1985-01

**Authors:** C. Paraskeva, B. G. Buckle, P. E. Thorpe

## Abstract

**Images:**


*Br. J. Cancer* (1985), **51**, 131–134

**Short Communication**

### Selective killing of contaminating human fibroblasts in epithelial cultures derived from colorectal tumours using an anti Thy-1 antibody-ricin conjugate

C. Paraskeva*, B.G. Buckle & P.E. Thorpe

*Imperial Cancer Research Fund, 44 Lincoln's Inn Fields, London WC2A 3PX, UK.*

We have recently developed a culture system for the growth of epithelial cells from both benign tumours (adenomas) and carcinomas derived from the colorectum of Familial polyposis coli (FPC) patients with which we intend to study tumour progression *in vitro* (Paraskeva *et al.*, 1984). One of the problems that we encountered was that contaminating fibroblastic cells in the cultures tended to outgrow the epithelial cells even when the initial fibroblastic contamination was low. Several methods for dealing with this problem have been reported (Gilbert & Migeon, 1975; Rheinwald & Green, 1975; Edwards *et al.*, 1980) but none of them is universally applicable.

The Thy-1 antigen was originally described as a cell surface antigen on mouse thymus and brain cells (Reif & Allan, 1964), Homologous structures have since been detected on a number of different cell types including rat (Stern, 1973) and human fibroblasts (Cotmore *et al.*, 1981) but appear to be absent from normal colorectal epithelium and colorectal carcinoma cells (Daar & Fabre, 1983). This difference in the distribution of the antigen suggested that by coupling ricin to monoclonal anti-human Thy-1 antibody we would be able to prepare a selective cytotoxic agent for Thy-1 expressing cells and use it to rid epithelial cell cultures of fibroblastic cells.

All tumour specimens were obtained from St Mark's Hospital, London, UK. A detailed description of the conditions for growing and characterizing the epithelial cells has been reported previously (Paraskeva *et al.*, 1984). Briefly, tumour specimens were washed, minced with scissors and digested at 37°C with a solution of collagenase (Worthington, type 4, $1.5\,mg\,ml^{-1}$) and hyaluronidase (Sigma, type 1, $0.25\,mg\,ml^{-1}$) in Dulbecco's modified Eagles Medium (DMEM). After overnight incubation (~12–16h) with the enzymes the tissue had disintegrated into glandular epithelial tubules, clumps of cells, single cells and cell debris. The digest was filtered through a 50 micron nylon mesh filter. The glandular epithelial tubules and large clumps of cells were collected from the filter, washed and put into culture. The filtrate mostly contained cell debris and single cells and when cultured gave rise mostly to fibroblastic cells. Within seven days most of the glandular epithelial tubules and clumps had attached to the substratum and given rise to epithelial outgrowths that were contaminated to varying degrees with fibroblastic cells. The standard culture medium for the epithelium was DMEM supplemented with 20% Foetal Bovine Serum, hydrocortisone $1\,\mu g\,ml^{-1}$, insulin $0.2\,units\,ml^{-1}$, and glutamine 2 mM. Cells were cultured on 5 cm petri dishes that had previously been coated with a film of collagen type IV (human placental collagen, Sigma) in the presence of Swiss 3T3 feeder cells which had previously been killed with Mitomycin C (Paraskeva *et al.*, 1984).

Two normal adult human skin fibroblast cell lines BRO and REE and one foetal human fibroblast cell line HEL 119 were kindly provided by Dr A.M.R. Taylor, Department of Cancer Studies, Cancer Research Campaign Laboratories, The Medical School, Birmingham, B15 2TJ, UK. One fibroblast cell line, designated PC/JD, was isolated in this laboratory from a colorectal tumour specimen derived from a polyposis coli patient. Two epithelial cell lines, PC/AA and PC/FF were derived from the adenomas of familial polyposis coli patients and have been described in detail elsewhere (Paraskeva *et al.*, 1984).

Ricin, the toxin from the Castor bean was kindly provided in purified form by Dr J.A. Forrester, Institute of Cancer Research, London, U.K. The monoclonal antibodies F-15-42-1-5 against human Thy-1 and F-8-11-13 against part of the human leukocyte common antigen were kindly provided as purified IgG by Dr J. Fabre, East Grinstead Hospital, Surrey, UK. Details of the origin of the

Correspondence: C. Paraskeva
*Present address: Department of Pathology, University of Bristol, The Medical School, University Walk, Bristol BS8 1TD, U.K.
Received 12 September, 1984

© The Macmillan Press Ltd., 1985

hybridomas and of the specificity of the antibodies that they produce have been previously published (McKenzie & Fabre, 1981; Dalchau & Fabre, 1981). The antibody-ricin conjugates were kindly provided by Prof. W. Ross and Dr A. Cumber of the Institute of Cancer Research, London, U.K. They had been prepared using the N-hydroxysuccinimidyl ester of chlorambucil as the coupling agent as described by Thorpe & Ross, 1982. The component of the conjugated products that correspond to one molecule of ricin linked to one molecule of antibody were isolated by gel filtration and characterised as reported previously (Thorpe & Ross, 1982).

An isotonic (270 mM) solution of galactose in distilled water was diluted with standard growth medium to a final galactose concentration of 100 mM ("galactose medium"). The stock solution of antibody-ricin conjugate contained 0.110 mg ricin $ml^{-1}$ and was diluted in galactose medium to give a final concentration of $10^{-3}$ mg ricin $ml^{-1}$. Medium was removed from the cultures under test, the cells washed once and 2.5 ml of galactose medium containing the antibody-ricin conjugate added and the cultures returned to the incubator. After one hour the conjugate was removed by washing the cells three times in galactose medium and then three times in standard growth medium. The purpose of the galactose in the incubation and washing media was to block non-specific toxicity to epithelial cells which otherwise would have resulted from the binding of the conjugate through its ricin moiety to galactose residues on the surface of the cells.

Preliminary experiments using a range of antibody-ricin conjugate concentrations established that the conjugate at $10^{-3}$ mg ricin $ml^{-1}$ was effective at killing both normal adult and foetal fibroblasts, and fibroblasts derived from familial polyposis coli patients, but showed no detectable toxicity towards the epithelial cell line PC/AA. This concentration was therefore chosen for all subsequent work. Three normal human fibroblast cell lines, BRO, REE, HEL 119 and one fibroblast line, PC/JD, derived from a polyposis coli patient were inoculated at $2.5 \times 10^5$ cells per 5 cm petri-dish. Twenty-four hours later they were treated with the antibody-ricin conjugate as described above. Control dishes were treated with galactose medium alone. Within four to seven days virtually all the conjugate-treated fibroblasts had degenerated and had detached from the culture dish, whereas the control fibroblasts were confluent (see Figure 1 for a typical result). The conjugate was effective on both confluent and subconfluent fibroblastic cultures, growing either on plastic or collagen coated petri-dishes. Any fibroblasts that survived the first treatment were destroyed by further treatment with the conjugate. The conjugate showed no detectable toxicity towards the epithelial cell lines PC/AA and PC/FF (Figure 1; PC/FF is not shown). Subcultures of epithelium treated with the conjugate showed no detectable difference in growth rate or survival from those treated with galactose alone, again indicating that the conjugate had not inflicted toxicity towards the epithelial cells.

The conjugate was used routinely to rid primary epithelial cell cultures of fibroblasts without any detectable toxicity towards the epithelial cells. Figure 2(a) shows a primary culture of epithelial cells heavily contaminated with fibroblasts. The culture was derived from a colorectal adenocarcinoma from a polyposis coli patient. Because the colony shown in Figure 2(a) was so heavily contaminated it required four treatments with the

**Figure 1** Cells ($2.5 \times 10^5$) of the adenoma derived cell line PC/AA were inoculated onto 5 cm petri-dishes. Six days later the cells were treated with (1) galactose medium control (2) standard medium control (3) anti Thy-1 antibody-ricin conjugate. Fourteen days after the treatment cultures were fixed in Formalin and stained with methylene blue. Cells ($2.5 \times 10^5$) of the human fibroblast cell line REE were inoculated onto 5 cm petri dishes. Twenty-four hours later the cultures were treated with (4) galactose medium control (5) anti Thy-1 antibody-ricin conjugate. Seven days after the treatment cultures were fixed in formalin and stained with methylene blue.

**Figure 2** (a) Primary culture derived from a colorectal adenocarcinoma showing an epithelial colony heavily contaminated with fibroblasts. The arrow shows the margin of the epithelial colony ( x 150). (b) Same area of culture as in 2(a) four weeks later after 4 treatments (at approximately weekly intervals) with the anti Thy-1 antibody-ricin conjugate. In this particular culture 3T3 feeders were not used so as not to confuse them with contaminating fibroblasts.

conjugate at approximately weekly intervals to kill all the fibroblastic cells. Figure 2(b) shows the same part of the epithelial colony four weeks after the antibody-ricin conjugate treatment had started. The two remaining fibroblastic cells in the field of view showed no mitotic activity, and no regrowth of fibroblasts was observed in subsequent subcultures. Passaging the cells appeared to facilitate the killing of fibroblasts, probably by dispersing any clumps or very dense areas of fibroblasts or by releasing cells hidden beneath the epithelium.

To check that the cytoxic effect of the anti-Thyl-ricin conjugate upon the fibroblasts was mediated through its binding to antigens on their surface, a second conjugate was prepared from an antibody (F-8-11-13) that reacted with neither fibroblasts nor epithelial cells. No toxicity towards either cell type was observed when the control conjugate was used under the same conditions as described above for the anti-Thyl-ricin conjugate (results not shown).

Various methods have previously been described for the removal of contaminating fibroblasts from epithelial cultures. EDTA has been reported to be very effective at selectivity removing contaminating human fibroblasts from human keratinocyte cultures (Rheinwald & Green, 1975). However, when tested in our system we found EDTA to detach the epithelial cells as efficiently as the fibroblasts (results not shown). Others have reported the use of a fibroblast-specific monoclonal antibody and complement to lyse fibroblasts but found that complement was somewhat toxic to the epithelium on its own (Edwards *et al.,* 1980). Another disadvantage of complement is that its activity can vary with each batch prepared. In the present study, an anti-Thyl-ricin conjugate was found to provide a simple and efficient means of selectively killing fibroblastic cells in culture. With its use, pure epithelial cell cultures and cell lines could routinely be established even when only a small amount of epithelium was available to initiate cultures, as is often the case with human tissue specimens. An advantage of the technique was that the epithelium could be set up untreated in primary culture and allowed to expand in cell number before killing fibroblastic colonies with the conjugate. This is of particular value in the early period of culture, during which establishment of certain epithelial cell lines may be dependent on mesenchymal-epithelial cell interactions.

We thank Dr W.F. Bodmer for suggesting the use of the anti Thy-1 antibody-ricin conjugate to selectively kill contaminating fibroblasts.

### References

COTMORE, S.F., CROWHURST, S.A. & WATERFIELD, M.D. (1981). Purification of thy-1-related glycoproteins from human brain and fibroblasts: comparison between these molecules and murine glycoproteins carrying Thy-1.1 and Thy-1.2 antigens. *Eur. J. Immunol.*, **11**, 597.

DAAR, A.S. & FABRE, J.W. (1983). The membrane antigens of Human Colorectal Cancer Cells: Demonstration with monoclonal antibodies of heterogeneity within and between tumours and of anomalous expression of HLA-DR. *Eur. J. Cancer Clin. Oncol.*, **19**, 209.

DALCHAU, R. & FABRE, J.W. (1981). Monoclonal antibody to a B lymphocyte specific determinant of the human leucocyte common antigen. *J. Exp. Med.*, **153**, 753.

EDWARDS, P.A.W., EASTY, D.M. & FOSTER, C.S. (1980). Selective culture of epitheliod cells from a human squamous carcinoma using a monoclonal antibody to kill fibroblasts. *Cell. Biol. Int. Rep.*, **4**, 917.

GILBERT, S.F. & MIGEON, B.R. (1975). D-valine as a selective agent for normal human and rodent epithelial cells in culture. *Cell*, **5**, 11.

McKENZIE, J.L. & FABRE, J.W. (1981). Restriction of Thy-1 to areas of rapid lymphocyte recirculation in human lymphoid tissues. *J. Immunol.*, **125**, 843.

PARASKEVA, C., BUCKLE, B., SHEER, D. & WIGLEY, C.B. (1984). The isolation and characterization of colorectal epithelial cell lines at different stages in malignant transformation from familial polyposis coli patients. *Int. J. Cancer*, **34**, 49.

REIFF, A.E. & ALLEN, J.M.V. (1964). The AK 12 thymic antigen and its distribution in leukaemias and nervous tissue. *J. Exp. Med.*, **120**, 413.

RHEINWALD, J.G. & GREEN, H. (1975). Serial cultivation of strains of human epidermal keratinocytes: the formation of keratinizing colonies from single cells. *Cell*, **6**, 331.

STERN, P.J. (1973). Allo antigen on mouse and rat fibroblasts. *Nature*, (*New Biol.*), **246**, 76.

THORPE, P.E. & ROSS, W.C.J. (1982). The preparation and cytotoxic properties of antibody-toxin conjugates. *Immunol. Rev.*, **62**, 119.